# Introduction

**DOI:** 10.1155/S0962935195000767

**Published:** 1995-05

**Authors:** Paul Van Cauwenberge

**Affiliations:** 1 Department of Otorhinolaryngology University of Gent Belgium


Mediators of Inflammation 4, 456 (1995)

### Book review

### Raeburn D, Giembycz MA. *Airways smooth muscle: Structure, Innervation and Neurotransmission.* Birkhäuser Verlag, Basel, 1994.

This book is the first of a new series on respiratory pharmacology and pharmacotherapy, and is part of a subseries of monographs on airway smooth muscle which will comprise three volumes: part two will cover development and regulation of contractility, and part three will cover biochemical control of contraction and relaxation.

The present 328 page volume offers a comprehensive overview of airway smooth muscle structure and neural regulation. It contains 12 chapters on anatomy and on the role of the different components of the autonomic innervation. All contributions come from recognized experts and most of these have succeeded in presenting their topic in a clear, concise way and in a pleasant style. All chapters have an extensive reference list, often up-to-date until 1993, sometimes even 1994.

The anatomy is covered in three chapters, an overview by Gabella, a chapter on immunocytochemistry of peptidergic nerves by Springall and Polak and a chapter on neural elements by the Laitinens. The sympathetic nervous system is discussed by Ind, and the parasympathetic system by Canning and Undem. Not surprisingly, a lot of space is devoted to non-adrenergic, noncholinergic innervation with chapters on excitatory peptides (Karlsson), inhibitory peptides (Uddman *et al.*) and nitric oxide (Belvisi and Bai). A chapter by Barnes summarizes factors that modulate neurotransmitter release in health and disease. Widdicombe and Wells critically describe the role of vagal reflexes.

There is often overlap between different chapters. Most authors include a description of the ultrastructure of 'their' part of the innervation, which can also be found in chapters on structure. For obscure reasons the chapter on anatomy of neural elements concludes the book, while it would have fitted nicely after the chapter on smooth muscle anatomy and ultrastructure, preceding the chapters on functional aspects. In the chapter on muscle structure, there are six pages on neural elements which are clearly redundant. It may be confusing that data from animals and man are mixed up in most chapters. Sometimes, bits of information are grouped according to the species in which they were obtained, e.g. in the chapter on NO, but more often the species are mixed under headings that refer to function or structure. The notion that animals may be entirely different from each other and from humans with respect to their autonomic regulation of airway smooth muscle is not always evident; this is where a chapter on differences between species would have been useful.

Another point of criticism is the quality of the picture illustrations. Most micrographs require considerable effort to distinguish the important details and marking letters are often almost impossible to discern. Glossy paper would have done better for EM pictures and in a book of this price light micrographs should have been printed in full colour.

Apart from all abundant and generally excellent information, the book misses a general perspective. It starts without a preface and, after 321 pages, leaves the reader with this final sentence: 'However, a dense network of VIP-immunoreactive nerve fibers has been found to be scattered around the nerve cell bodies of these microganglia.' This may not be disturbing for insiders who just wish a reference guide, but will disappoint the interested chest physician or beginning scientist who may have some difficulty in putting things together. An introductory chapter briefly reviewing the relevant issues, their coherence and their historical background is missing. Nowhere in the book is it stated that two more volumes will follow to cover other aspects of airway smooth muscle.

In summary, this book offers a lot of high-quality information in a comprehensive format, and is an excellent reference guide to recent literature. It can be recommended for those who want to start research on airway smooth muscle, for the interested clinician and for those already working in the field who will appreciate a summary of recent research. The relatively high price will probably be an obstacle for buying a personal copy, but university libraries should acquire this promising first part of what could become a valuable state-of-the-art series on a topic that is of utmost importance for human respiratory health and disease.

REVIEWER

Johan C. de Jongste MD, PhD,
Erasmus University and University Hospital/Sophia Children's Hospital Rotterdam, the Netherlands.

© 1995 Rapid Science Publishers

